# Low IL7R Expression at Diagnosis Predicted Relapse in Adult Acute Myeloid Leukemia Patients With t(8;21)

**DOI:** 10.3389/fimmu.2022.909104

**Published:** 2022-07-07

**Authors:** Nan Xu, Kai Sun, Ya-Zhe Wang, Wen-Min Chen, Jun Wang, Ling-Di Li, Xu Wang, Yue Hao, Yan Chang, Yan-Rong Liu, Xiao-Jun Huang, Ya-Zhen Qin

**Affiliations:** Peking University People’s Hospital, Peking University Institute of Hematology, National Clinical Research Center for Hematologic Disease, Beijing, China

**Keywords:** t(8;21) AML, IL7R, relapse, real-time quantitative PCR, RNAseq, flow cytometry

## Abstract

**Background:**

Acute myeloid leukemia (AML) with t(8;21) needs to be further stratified. In addition to leukemia cells, immune cells in tumor microenvironment participate in tumor initiation, growth and progression. Interleukins (ILs)/interleukin receptors (ILRs) interaction plays important roles in the antitumor immune response. IL7R is reported to be relevant to prognosis in solid tumor and acute lymphoblastic leukemia. However, the prognostic significance of IL7R in t(8;21) AML remains to be clarified.

**Methods:**

Bone marrows collected from 156 newly diagnosed t(8;21) AML patients were used for testing IL7R transcript level by TaqMan-based real-time quantitative PCR (RQ-PCR), and RNAseq were performed in 15 of them. Moreover, IL7R expression at diagnosis were measured by RQ-PCR and flow cytometry (FCM) simultaneously in other 13 t(8;21) AML patients.

**Results:**

t(8;21) AML patients had varied IL7R transcript levels and were categorized into low-expression (IL7R-L) and high-expression (IL7R-H) groups; IL7R-L was significantly associated with a lower relapse-free survival (RFS) rate (*P*=0.0027) and KIT^D816/D820^ mutation (*P*=0.0010). Furthermore, IL7R-L was associated with a lower RFS rate in KIT^D816/D820^ group (*P*=0.013) and IL7R-H/KIT^D816/D820^ patients had similar RFS to KIT^N822/e8/WT^ patients (*P*=0.35). GO analysis enrichment showed that down-regulated genes were predominantly involved in the regulation of T cell and leukocyte activation, proliferation and differentiation in IL7R-L group. IL7R-L had significantly lower levels of Granzymes A/B, CCR7, CD28 and CD27 than IL7R-H group (all *P*<0.05). FCM analysis showed IL7R protein was primarily expressed in CD4^+^ T and CD8^+^ T cell subset. A significant association was found between the transcript level of IL7R and the percentage of CD8^+^ T cells in nucleated cells (*P*=0.015) but not CD4^+^ T cells (*P*=0.47).

**Conclusion:**

Low IL7R transcript level of bone marrow at diagnosis predicted relapse in t(8;21) AML, which might be caused by the difference in the amount, status and function of T cells.

## Introduction

Acute myeloid leukemia (AML) with t(8;21) is a heterogeneous hematological malignancy, although it was categorized into a favorable-risk group (1–3), 30–40% of patients relapse (4, 5). At present, the well-established RUNX1-RUNX1T1 transcript level and c-KIT mutation status at diagnosis are the main prognostic factors in t(8;21) AML (6–9). However, they do not accurately stratify patients. Therefore, new prognostic biomarkers for patients with t(8;21) AML are needed in order to improve risk stratification and guide precise treatments.

In addition to leukemia cells themselves, cells of the immune system are a fundamental component of the tumor microenvironment (TME), which often modify the TME to be more favorable to tumor development and progression through producing cytokines and mediators (10, 11). Interleukins (ILs)/interleukin receptors (ILRs) interaction plays important roles in the antitumor immune response through mediating cell–cell communication in TME and is reported to be relevant to patient prognosis (12–14).

As a member of the Interleukin family, Interleukin 7 (IL7) play vital roles in hematopoiesis and the development of T lymphocytes, as well as the inflammation, autoimmune diseases and hematological cancers. Its function is mediated by the IL7 receptor, which is a membrane receptor consisted of the specific IL7Rα chain (referred as IL7R) and IL-2Rγ chain (common gamma chain shared by receptors for other cytokines) (15–17). IL7R is mainly expressed by cells of the lymphoid lineage, especially in both naïve and memory T cells. It is reported that IL7R was amplified in different solid cancers, including breast and lung cancer, and its high level was associated with poor prognosis (18–20). So far, researches of IL7R in hematological malignancies have been mainly focused on acute lymphoblastic leukemia (ALL) and lymphoma, in which the prognostic impact was contradictory; several studies reported that IL7R is highly expressed and an upregulation of IL7R may predict relapse (21, 22), whereas Cleaver et al. demonstrated that low transcript expression of IL7R was significantly related to relapse (12). Furthermore, report on the prognostic role of IL7R expression in AML remains absent to date.

In the present study, by retrospectively testing IL7R transcript of 156 adult t(8;21) AML patients who were consecutively treated at our institute, we comprehensively explored its impact on relapse. We further explored the mechanism of prognostic impact through the enrichment of gene expression data from RNAseq. Moreover, we evaluated the expression pattern of IL7R protein in different cell subsets through flow cytometry.

## Materials and Methods

### Patients and Treatment

A total of 156 adult t(8;21) AML patients who were consecutively diagnosed, received treatment and achieved complete remission (CR) at our institute between January 2013 and January 2020 were included. The median age of the patients at diagnosis was 38 (range: 15–67) years, and 80 (51.3%) of them were male. The diagnosis of t(8;21) AML was made according to bone marrow morphology, immunophenotyping, karyotyping, and molecular biology. RUNX1-RUNX1T1 transcript level of bone marrow were measured by real-time quantitative polymerase chain reaction (RQ-PCR) at diagnosis and during treatment for minimal residual disease (MRD) monitoring as we described previously (23). c-KIT mutations in exons 17 and 8 were screened at diagnosis through bidirectional Sanger sequencing (8).

As we previously reported (9, 24), the patients received induction chemotherapy composed of 1–2 cycles of an anthracycline in combination with cytarabine or the HAA regimen (homoharringtonine, aclarubicin, and cytarabine). After achieving CR, patients received cytarabine-based chemotherapy alone or chemotherapy followed by allogeneic HSCT (allo-HSCT) as postremission therapy. The cutoff date for follow-up was November 2021. This research was approved by the Ethics Committee of Peking University People’s Hospital and carried out in accordance with the Helsinki Declaration.

### Detection of IL7R Transcript Level

IL7R transcript levels were retrospectively detected in bone marrow samples collected from all patients at diagnosis. RNA was extracted from bone marrow nucleated cells to synthesize complementary DNA. IL7R transcript level were measured by TaqMan-based RQ-PCR technology. The primers and probe for the IL7R transcript were designed using primer design program, Primer3 (25), and the sequences were as follows: forward primer: 5’-GATGTAGCTTACCGCCAGGA-3’; reverse primer: 5’-CCATTCACTCCAGAAGCCTTT-3’; probe: 5’-FAM-AAGCTGACACTCCTGCAGAGAAAGCT-TAMRA-3’. ABL was selected as a control gene for normalization (26). Quantification of IL7R transcript level was conducted using the 2^−ΔCT^ method and expressed in percentage.

### RNA-Seq and Gene Ontology (GO) Analysis

Total RNA extracted from bone marrow samples collected at diagnosis of 15 t(8;21) AML patients were performed sequencing on Illumina NovaSeq 6000 platform. RNA sequence reads were aligned to the human genome (hg38) using Hisat2 v2.0.5. Gene reads were counted by featureCounts v1.5.0-p3. Fragments Per Kilobase of transcript sequence per Millions (FPKM) of each gene was calculated based on the length of the gene and reads count mapped to this gene. Fold changes were computed as the log2 ratio of normalized reads per gene using DEseq2 package in R software (version 3.6.2). Genes expression with |log2 (fold change)| ≥1.0 and adjusted P values <0.05 were considered as diferentially expressed genes (DEGs). GO analysis was performed based on clusterProfiler, enrichplot, org.Hs.eg.db and ggplot2 packages in R software.

### Flow Cytometry Analysis

Multi-color Flow cytometry (FCM) experiment were carried out on bone marrow samples collected from 13 newly diagnosed t(8;21) AML patients from March to May 2021 and performed using FACSCanto™ II (BD Biosciences, San Jose CA, USA). The monoclonal antibodies included CD127(IL7R)-APC (Biolegend, Clone A019D5), CD45-V500 (BD Biosciences, Clone HI30), CD19-BV605 (Biolegend, Clone HIB19), CD34-FITC (BD Biosciences, Clone 8G12), CD56-APC-F750 (Biolegend, Clone 5.1H11), CD3-BV421 (Biolegend, Clone OKT3), CD4-PE (Caprico Biotechnologies, Clone OKT4), CD8-PE-Cy7 (BD Biosciences, Clone SK1).

The gating strategy is shown in **Figure S1**. The FSC-H/FSC-A plot was used to exclude doublets. The FSC-A/SSC-A plot was performed to remove cellular debris and nonviable cells and defined as nucleated cells (NCs). Lymphocytes were defined as CD45-high/SSC-A-low. T, B and NK cells were defined as CD3^+^, CD3^-^CD56^-^ and CD3^-^CD56^+^ from lymphocytes, respectively. CD4^+^ T and CD8^+^ T cells were derived from CD3^+^ cells. In parallel, AML blast and myeloid cells (abbreviated as Blast+Mye) were defined as CD45-dim/SSC-A-low. CD34^+^ and CD34^-^ cells were defined in CD34/SSC-A plot. Expression of CD127 was assessed on CD4^+^ T, CD8^+^ T, B, NK, and CD34^+^ Blast+Mye as well as CD34^-^ Blast+Mye cells, respectively. Positive gates for IL7R were determined based on isotype control stains.

### Statistical Analysis

The Mann–Whitney U test was used for the pairwise comparisons of continuous variables between groups and Fisher’s exact test for categorical variables. The bivariate Spearman correlation test was used to measure associations among continuous variables. A receiver operating characteristic (ROC) curve was used to identify the optimal cutoff level that best discriminated patients with relapse. Relapse-free survival (RFS) was measured from the date when CR was achieved to the date of last bone marrow evaluation. Overall survival (OS) was defined as time from diagnosis to death or last follow-up. The events were relapse for RFS and death for OS (regardless of the cause), and patients were queried at the date of last follow-up to determine whether they were still alive, or were censored on the date they were last known to be alive. Survival functions were estimated using the Kaplan-Meier method and compared using the log-rank test. The level for a statistically significant difference was set at *P*<0.05 for all univariate tests. Variables associated with *P*<0.20 in the univariate analysis were entered into multivariate analysis which performed by the Cox models. The SPSS 26.0 software package (SPSS Inc., Chicago, IL), and GraphPad Prism 8 (GraphPad Software Inc., La Jolla, CA) were used for data analysis.

## Results

### Patients’ Characteristics and Outcomes

Of 156 t(8;21) AML patients included, 138 (88.5%), 15 (9.6%) and 3 (1.9%) patients individually achieved CR after one, two and three cycles of induction chemotherapy. Thereafter, 101 patients received chemotherapy alone and 3 and 52 individually received 1 and ≥ 2 courses of chemotherapy followed by allo-HSCT at CR1 status (matched sibling donor, n=16; haploidentical related donor, n=38; matched unrelated donor, n=1). 39 patients (25.0%) experienced hematological relapse, and 127 patients (81.4%) were alive at the last follow-up. The median follow-up time was 36.0 (range, 5.8–106.5) months for the entire cohort. The 4-year RFS and OS rate were 71.4% [95% confidence interval (CI), 62.3–78.7%] and 79.1% (95% CI, 70.7–85.4%), respectively.

### Transcriptional Expression Pattern of IL7R in t(8;21) AML Patients at Diagnosis

**Figure 1:**
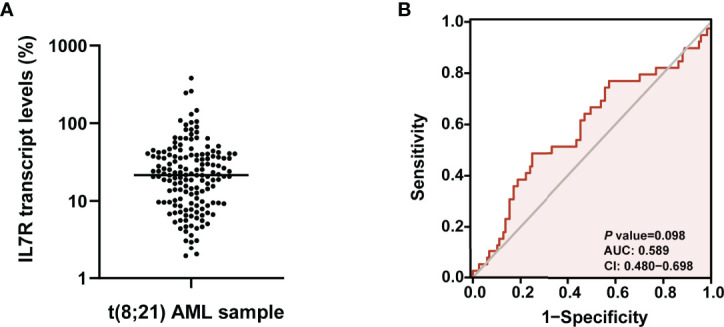
**(A)** The expression pattern of IL7R transcript level in bone marrow of t(8;21) AML patients at diagnosis; **(B)** ROC curve analysis based on relapse were performed to determine the optimal cutoff value of IL7R transcript level for grouping.

The median IL7R transcript level of all 156 patients at diagnosis was 21.6% (range: 1.9-380.2%, **Figure 1A**). ROC curve analysis based on relapse were performed (**Figure 1B**), and 13.0% had the maximal Youden index (0.239) among all values [area under curve (AUC)=0.589, *P*=0.098]. Thus, 13.0% was the optimal cutoff value which categorized patients into 2 groups: higher than 13.0% (n=108, 69.2%) and the rest (n=48, 30.8%) which were defined as IL7R-H and IL7R-L, respectively.

### The Impact of IL7R Transcript Level at Diagnosis on Relapse

**Figure 2:**
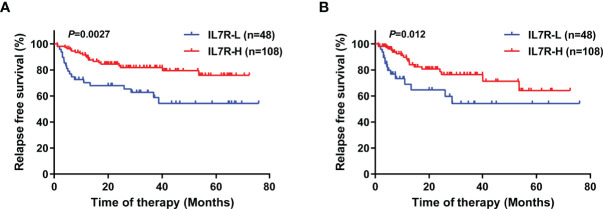
The impact of IL7R transcript level at diagnosis on relapse in t(8;21) AML patients using Kaplan–Meier survival analysis (log-rank test). **(A)** RFS, with no censoring; **(B)** RFS, with censoring at the time of allo-HSCT.

IL7R-L patients had significantly lower RFS rate than IL7R-H patients (4-year RFS: 54.3% [95% CI, 39.6-69.0%] vs 79.5% [95% CI, 69.0-86.7%], *P*=0.0027, **Figure 2A**). Similar results existed if patients who received allo-HSCT were censored at the time of transplantation (4-year RFS: 54.3% [95% CI, 33.5-71.0%] vs 71.3% [95% CI,55.1-82.5%], *P*=0.012, **Figure 2B**). Whereas, IL7R-L had a similar OS rate to IL7R-H patients (4-year OS: 74.5% [95% CI, 57.0-85.7%] vs 81.0% [95% CI, 70.8-88.0%], *P*=0.27).

### Relationship Between IL7R Expression at Diagnosis and Other Patients’ Pre-Treatment Characteristics and MRD

Overall, 64 patients (41.0%) had c-KIT mutations at diagnosis. Based on our previous study (27), c-KIT mutation status was categorized into D816/D820 mutation (abbreviated as KIT^D816/D820^, n=35) and N822/e8/WT (abbreviated as KIT^N822/e8/WT^, n=121) groups. According to our prior reports (9, 28), 0.4% (3-log reduction from the pretreatment baseline level) of the RUNX1-RUNX1T1 transcript was selected as the cutoff value for MRD after 2nd consolidation chemotherapy. Thus, >0.4% and ≤0.4% of RUNX1-RUNX1T1 transcript level after 2nd consolidation chemotherapy were individually defined as a high or low MRD level status (abbreviated as MRD-H and MRD-L). Totally, 143 patients received at least 2 courses of consolidation therapy, and 43 and 100 of them were identified as MRD-H and MRD-L, respectively.

As shown in **Table 1**, IL7R-L was significantly related to higher white blood cell (WBC) counts (*P*=0.015), higher bone marrow blast percentage (*P*=0.042), MRD-H (*P*=0.0060) and KIT^D816/D820^ (*P*=0.0010). Whereas, it was not related to age, sex, hemoglobin levels, platelet counts and karyotypes (all *P*>0.05).

**Table 1:** Relationship between IL7R expression at diagnosis and other characteristics.

Variable	All	IL7R transcript level		*P* value
		IL7R-L	IL7R-H	
Number of patients	156	48	108	
Age (year, median, range)	38 (14-67)	35 (15-60)	39 (14-67)	0.50
Males (%)	80 (51.3%)	26 (54.2%)	54 (50.0%)	0.73
WBC count (×10^9^/L; median; range)	8.7 (0.7-101.0)	12.7 (1.2-59.2)	7.5 (0.7-101.0)	0.015
Hemoglobin (g/L; median; range)	76 (31-151)	79 (38-129)	76 (31-151)	0.87
Platelet count (×10^9^/L; median; range)	31 (3-324)	30 (5-171)	33 (3-324)	0.098
Bone marrow blast (%, median, range)	48 (8-91)	55 (12-91)	46 (8-90)	0.042
Patients with cytogenetic abnormalities other than t(8;21), n (%)(n=152)				
No	64	20 (41.7%)	44 (42.3%)	1.0
Yes	88	28 (58.3%)	60 (57.7%)	
KIT gene mutation status, n (%)				
D816/D820	35	19 (39.6%)	16 (14.8%)	0.0010
N822/e8/WT	121	29 (60.4%)	92 (85.2%)	
MRD status, n (%)(n=144)				
MRD-L	101	23 (53.5%)	78 (77.2%)	0.0060
MRD-H	43	20 (46.5%)	23 (22.8%)	

### Univariate and Multivariate Analysis of RFS

Univariate analysis was performed and the results were shown in **Table 2**. In addition to IL7R-L, KIT^D816/D820^, older age, treating with chemotherapy alone and MRD-H were all significantly associated with lower RFS rate (all *P<*0.05). Whereas, sex, WBC count, hemoglobin level, platelet count, bone marrow blast percentage and karyotypes had no impact on relapse (all *P*>0.05). The multivariate analysis showed that KIT^D816/D820^, treating with chemotherapy alone and MRD-H were independent adverse prognostic factors for RFS in t(8;21) AML patients (hazard ratio [HR]=7.4 [95% CI 3.0-17.9], *P*<0.0010; HR=17.7 [95% CI 5.5-56.5], *P*<0.0010; HR=5.1 [95% CI 2.3-11.0], *P*<0.0010). However, IL7R transcript level was not an independent prognostic factor for RFS (*P=*0.28).

**Table 2:** Univariate and multivariate analysis of relapse.

Variables	No. of patients	Univariate analysis		multivariate analysis
		4-year RFS rate (95%CI)	*P* value	*P* value
IL7R transcript level				
≤13%	48	54.3% (36.7-69.0%)	0.0027	0.28
>13%	108	79.5% (69.0-86.7%)		
Age				
≤40	91	79.9% (68.8-87.4%)	0.016	0.093
>40	65	57.6% (40.2-71.5%)		
Sex				
Male	80	77.8% (65.7-86.0%)	0.21	–
Female	76	65.0% (50.3-76.3%)		
WBC count				
≤9×10^9^	79	71.3% (57.1-81.5%)	0.89	–
>9×10^9^	76	71.1% (57.9-80.9%)		
Hemoglobin				
≤80g/L	83	68.9% (54.8-79.5%)	0.99	–
>80g/L	70	72.3% (58.6-82.1%)		
Platelet count				
≤30×10^9^	76	69.0% (54.9-79.5%)	0.66	–
>30×10^9^	79	73.0% (59.7-82.5%)		
Bone marrow blast				
≤50%	87	70.1% (57.1-79.9%)	0.72	–
>50%	64	73.1% (58.9-83.0%)		
Patients with cytogenetic abnormalities other than t(8;21) (n=152)				
No	64	71.9% (56.7-82.6%)	0.89	–
Yes	88	71.7% (58.9-81.1%)		
KIT mutation status				
D816/D820	35	47.9% (28.9-64.7%)	<0.0001	<0.0010
N822/e8/WT	121	78.1% (67.7-85.5%)		
Treatment modality				
Allo-HSCT	55	88.3% (73.5-95.1%)	0.0002	<0.0010
Chemotherapy alone	101	61.7% (49.2-72.0%)		
MRD status				
MRD-L	43	81.1% (69.1-88.8%)	0.0027	<0.0010
MRD-H	101	62.2% (44.7-75.6%)		

### Impact of the Combination of IL7R Transcript Expression and c-KIT Mutation Status at Diagnosis on Relapse

In order to further explore the prognostic impact of IL7R transcript level, we combined it with c-KIT mutation status at diagnosis. As a result, 156 patients were categorized into the following 4 groups: IL7R-L/KIT^D816/D820^ (n=19, 12.2%), IL7R-L/KIT^N822/e8/WT^ (n=29, 18.6%), IL7R-H/KIT^D816/D820^ (n=16, 10.3%), and IL7R-H/KIT^N822/e8/WT^ (n=92, 59.0%).

**Figure 3:**
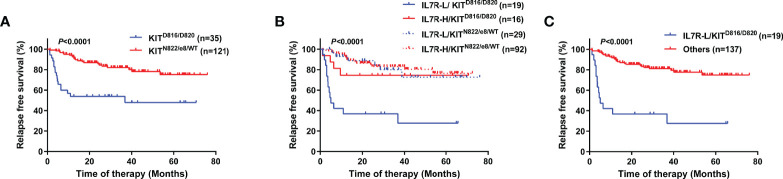
RFS of patients grouped by KIT or the combination of KIT and IL7R using Kaplan–Meier survival analysis (log-rank test). **(A)** Comparison between patients grouped by KIT mutation status; **(B)** Comparison between patients categorized into 4 groups; **(C)** Comparison between patients categorized into 2 groups.

KIT^D816/D820^ itself was significantly related to a lower RFS rate (4-year RFS, 47.9% [95% CI 28.8-64.7%] vs 78.1% [95% CI 67.7-85.5%], *P*<0.0001, **Figure 3A**). After combination, the 4 groups had significantly different RFS rate (*P*<0.0001, **Figure 3B**). IL7R-L/KIT^D816/D820^ group had a significantly lower RFS rate than IL7R-H/KIT^D816/D820^ group (4-year RFS, 27.6% [95% CI 8.8-50.6%] vs 74.5% [95% CI 45.4-89.6%], *P*=0.013), whereas there was no significant difference between IL7R-L/KIT^N822/e8/WT^ and IL7R-H/KIT^N822/e8/WT^ group (4-year RFS, 72.7% [95% CI 47.2-87.3%] vs 80.4% [95% CI 68.7-88.1%], *P*=0.73). Furthermore, IL7R-H/KIT^D816/D820^ group had similar RFS rate to KIT^N822/e8/WT^ group (4-year RFS, 74.5% vs 78.1%, *P*=0.35). Therefore, they were merged into one group which was named as Others. As shown in **Figure 3C**, IL7R-L/KIT^D816/D820^ group had a significantly lower RFS rate compared with Others group (4-year RFS, 27.6% [95% CI 8.8-50.6%] vs 77.6% [95% CI 68.1-84.7%], *P*<0.0001). After combination, 45.8% of patients (16/35) with KIT^D816/D820^ were recategorized from KIT-mutation-defined high-risk into low-risk group.

### GO Analysis and Comparison of Key Molecules Between IL7R-L and IL7R-H Group

**Figure 4:**
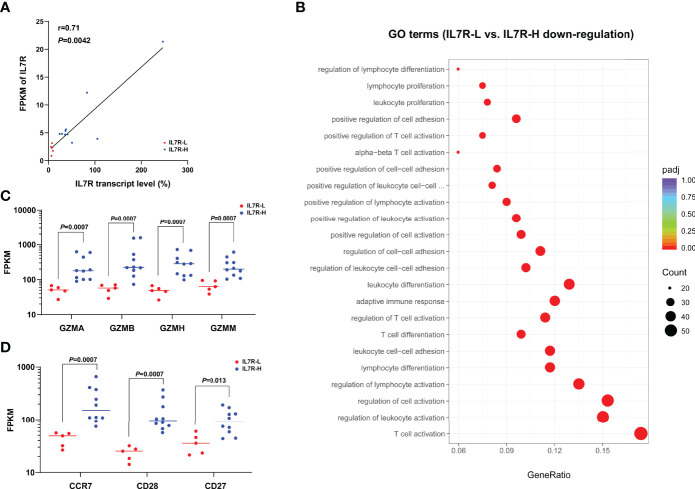
**(A)** Correlation of IL7R transcript level and FPKM of IL7R (bivariate Spearman correlation test); **(B)** Significantly down-regulated GO terms for IL7R-L compared with IL7R-H group; **(C)** Comparison of FPKM of granzymes A/B/H/M between IL7R-L and IL7R-H group; **(D)** Comparison of FPKM of CCR7, CD28 and CD27 between IL7R-L and IL7R-H group.

RNA samples from 5 IL7R-L and 10 IL7R-H patients were used to perform RNAseq. Their RQ-PCR-tested IL7R transcript level were significantly related to FPKM of IL-7R (r=0.71, *P*=0.0042, **Figure 4A**). IL7R-L patients had 454 down-regulated genes and 59 up-regulated genes compared with IL7R-H patients. As shown in **Figure 4B**, GO analysis enrichment showed that down-regulated genes were predominantly involved in the regulation of T cell and leukocyte activation, proliferation and differentiation in IL7R-L group. Furthermore, FPKM of granzymes A/B/H/M and the phenotypic markers of naïve and memory T cells (CCR7, CD28 and CD27) were significantly lower in IL7R-L group than that in IL7R-H group (all *P*<0.05, **Figures 4C, D**), respectively.

### Comparison of IL7R Tested by RQ-PCR and FCM

The IL7R transcript and its protein CD127 were simultaneously tested by RQ-PCR and FCM in other 13 bone marrow samples collected from t(8;21) AML patients at diagnosis, respectively. The CD127 expression was indicated as its frequency in nucleated cells (abbreviated as CD127/NCs). We found that IL7R transcript level was not significantly related to CD127/NCs (r=0.060, *P*=0.85).

**Figure 5:**
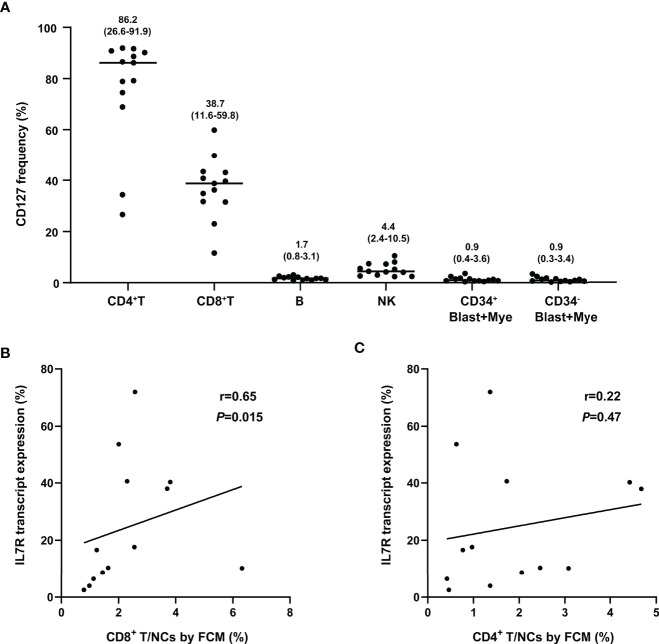
Comparison of IL7R tested by RQ-PCR and FCM. **(A)** The frequency of CD127 in different cell subsets, numbers on the figure represent median (range); **(B)** Correlation of IL7R transcript expression and percentage of CD8^+^ T in NCs (FCM) (bivariate Spearman correlation test); **(C)** Correlation of IL7R transcript expression and percentage of CD4^+^ T in NCs (FCM) (bivariate Spearman correlation test).

FCM analysis showed the expression pattern of CD127 in different cell subsets. As shown in **Figure 5A**, CD127 frequency was high in CD4^+^ T and CD8^+^ T cell subsets, low in NK cells and fairly low in blast, myeloid and B cells. Moreover, CD127 frequency was significantly higher on CD4^+^ T cells than that on CD8^+^ T cells (*P*=0.0005). Furthermore, a significant association was found between the transcript level of IL7R and CD8^+^ T/NCs but not CD4^+^ T/NCs (r=0.65, *P*=0.015; r=0.22, *P*=0.47, **Figure 5**).

## Discussion

Although AML with t(8;21) belongs to the favorable-risk group based on the current risk stratification strategies, its prognosis is quite variable (1–5). More prognostic indicators need to be identified in order to refine risk-adapted therapy.

In the current study, we found that bone marrows of newly diagnosed patients with t(8;21) AML had varied IL7R transcript levels. Furthermore, low IL7R transcript level at diagnosis was significantly associated with relapse. Multivariate analysis in the current study obtained the same results as we previously reported, that is, KIT^D816/D820^, treating with chemotherapy alone and MRD-H were independent adverse prognostic factors for RFS in t(8;21) AML patients (27). IL7R transcript level was not an independent prognostic factor for RFS and OS. We then combined IL7R transcript level with c-KIT mutation status for patient grouping. We found that low IL7R transcript level was significantly associated with a lower RFS rate in KIT^D816/D820^ group and about two fifths of them were recategorized into KIT^N822/e8/WT^ group in regard to relapse. Therefore, IL7R transcript level could be used to further differentiate patients with KIT^D816/D820^.

It has been found that IL7R is mainly expressed by cells of the lymphoid lineage and varies dramatically during the life of lymphoid cells (15, 29). It is expressed in B cell progenitors, but absent in mature B cells (30, 31). Likely, our multi-color flow cytometric analysis showed that CD127 was primarily expressed in CD4^+^ T and CD8^+^ T cell subsets, expressed low in NK cells and hardly expressed in blast, myeloid and B cells in bone marrow samples of t(8;21) AML patients. Therefore, IL7R transcript were mainly contributed by T cells in bone marrows. Several studies demonstrated that both naïve and memory T cells express high levels of IL7R, and IL7 is required for their homoeostasis (32–36). IL7R was absent on selecting thymocytes and effector cells (37, 38). In addition to IL7R (37, 38), CCR7, CD28 and CD27 are the phenotypic markers of naïve and memory T cells (39–41). Our RNAseq results showed that the expression of CCR7, CD28 and CD27 in the IL7R-L group were significantly lower than that in the IL7R-H group, which implied that IL7R-L patients had lower frequencies of naïve and memory T cells than IL7R-H patients. Xu et al. reported that AML patients had a decrease in stem cell memory and central memory CD8^+^ T cells together with an increase in differentiated CD8^+^ T effector memory and terminal effector cells compared with healthy individuals (42). Therefore, AML patients may have a decrease in memory cells and the degree of decrease impacted their outcomes.

CD8^+^ T lymphocytes are the major anti-tumor effector cells, and effectively enhancing the antitumor function of CD8^+^ T cells is the key to the treatment of tumors (43, 44). Granzymes A/B (GZMA/GZMB) are the key cytolytic effector molecules of CD8^+^ T and NK cells for killing target cells (45). In the current study, a significant association was found between IL7R transcript level and the percentage of CD8^+^ T cells in NCs but not CD4^+^ T cells. In addition, IL7R-L group had a significantly lower level of GZMA and GZMB than that of IL7R-H group. Moreover, GO analysis enrichment showed that down-regulated genes were predominantly involved in the regulation of T cell and leukocyte activation, proliferation and differentiation in IL7R-L group. Therefore, the prognostic significance of IL7R transcript level in t(8;21) AML might be mainly caused by T cells.

We found that IL7R transcript level was shown to be significantly related to KIT mutation status. c-KIT is primarily expressed on blast cells in t(8;21) AML. It is a type III receptor tyrosine kinase and its mutations lead to constitutive activation of the receptor causing an increased proliferation of cells during carcinogenesis (46, 47). Neoantigens derived from tumor-specific genetic mutations have high immunogenicity. Iiizumi et al. reported that immune responses to c-KIT-D816V were observed in more than 10% of the donors, which induced HLA class II-restricted CD4^+^ T cell responses and antigen-specific CD8^+^ T cells (48). Considering that it is KIT but not IL-7R that was an independent prognostic factor, we suspected that leukemic cells with KIT^D816/D820^ modified tumor microenvironment through affecting the profile of T cells.

In summary, low IL7R transcript level of bone marrow samples collected at diagnosis predicted relapse in patients with t(8;21) AML; patients with KIT^D816/D820^ could be further stratified by IL7R transcript level. Although the current study just detected IL7R transcript of bulk RNA, the survival analysis demonstrated its prognostic significance. Supported by the RNAseq and FCM results, the current study provides a clue on that the amount, status and function of T cells in bone marrow microenvironment may impact treatment outcomes of AML. The mechanism remains to be explored in depth in the future.

## Data Availability Statement

The data presented in the study are deposited in the NCBI Sequence Read Archive (SRA) repository, accession number PRJNA852777.

## Ethics Statement

The studies involving human participants were reviewed and approved by Ethics Committee of Peking University People’s Hospital. Written informed consent to participate in this study was provided by the participants’ legal guardian/next of kin.

## Author Contributions

Y-ZQ designed the study and revised the manuscript. NX analyzed data and wrote the manuscript. NX, W-MC, L-DL, XW, and YH performed the PCR. NX, KS, Y-ZW, JW, YC, and Y-RL performed flow cytometry. X-JH collected clinical data. All authors read the manuscript and gave the final approval.

## Funding

This work was supported by the National Nature Science Foundation of China (82070153).

## Conflict of Interest

The authors declare that the research was conducted in the absence of any commercial or financial relationships that could be construed as a potential conflict of interest.

## Publisher’s Note

All claims expressed in this article are solely those of the authors and do not necessarily represent those of their affiliated organizations, or those of the publisher, the editors and the reviewers. Any product that may be evaluated in this article, or claim that may be made by its manufacturer, is not guaranteed or endorsed by the publisher.

## References

[1] Marcucci G Mrozek K Ruppert AS Maharry K Kolitz JE Moore JO. Prognostic Factors and Outcome of Core Binding Factor Acute Myeloid Leukemia Patients with T(8;21) Differ from Those of Patients with Inv(16): A Cancer and Leukemia Group B Study. J Clin Oncol (2005) 23(24):5705–17. doi: 10.1200/JCO.2005.15.610 16110030

[2] Dohner H Estey E Grimwade D Amadori S Appelbaum FR Buchner T. Diagnosis and Management of Aml in Adults: 2017 Eln Recommendations from an International Expert Panel. Blood (2017) 129(4):424–47. doi: 10.1182/blood-2016-08-733196 PMC5291965 27895058

[3] Tallman MS Wang ES Altman JK Appelbaum FR Bhatt VR Bixby D. Acute Myeloid Leukemia, Version 3.2019, Nccn Clinical Practice Guidelines in Oncology. J Natl Compr Cancer Network: JNCCN (2019) 17(6):721–49. doi: 10.6004/jnccn.2019.0028 31200351

[4] Byrd JC Mrozek K Dodge RK Carroll AJ Edwards CG Arthur DC. Pretreatment Cytogenetic Abnormalities Are Predictive of Induction Success, Cumulative Incidence of Relapse, and Overall Survival in Adult Patients with *De Novo* Acute Myeloid Leukemia: Results from Cancer and Leukemia Group B (Calgb 8461). Blood (2002) 100(13):4325–36. doi: 10.1182/blood-2002-03-0772 12393746

[5] Schlenk RF Benner A Krauter J Buchner T Sauerland C Ehninger G. Individual Patient Data-Based Meta-Analysis of Patients Aged 16 to 60 Years with Core Binding Factor Acute Myeloid Leukemia: A Survey of the German Acute Myeloid Leukemia Intergroup. J Clin Oncol (2004) 22(18):3741–50. doi: 10.1200/JCO.2004.03.012 15289486

[6] Yin JA O’Brien MA Hills RK Daly SB Wheatley K Burnett AK. Minimal Residual Disease Monitoring by Quantitative Rt-Pcr in Core Binding Factor Aml Allows Risk Stratification and Predicts Relapse: Results of the United Kingdom Mrc Aml-15 Trial. Blood (2012) 120(14):2826–35. doi: 10.1182/blood-2012-06-435669 22875911

[7] Paschka P Marcucci G Ruppert AS Mrozek K Chen H Kittles RA. Adverse Prognostic Significance of Kit Mutations in Adult Acute Myeloid Leukemia with Inv(16) and T(8;21): A Cancer and Leukemia Group B Study. J Clin Oncol (2006) 24(24):3904–11. doi: 10.1200/JCO.2006.06.9500 16921041

[8] Qin YZ Zhu HH Jiang Q Jiang H Zhang LP Xu LP. Prevalence and Prognostic Significance of C-Kit Mutations in Core Binding Factor Acute Myeloid Leukemia: A Comprehensive Large-Scale Study from a Single Chinese Center. Leuk Res (2014) 38(12):1435–40. doi: 10.1016/j.leukres.2014.09.017 25449688

[9] Zhu HH Zhang XH Qin YZ Liu DH Jiang H Chen H. Mrd-Directed Risk Stratification Treatment May Improve Outcomes of T(8;21) Aml in the First Complete Remission: Results from the Aml05 Multicenter Trial. Blood (2013) 121(20):4056–62. doi: 10.1182/blood-2012-11-468348 23535063

[10] Hanahan D Weinberg RA. Hallmarks of Cancer: The Next Generation. Cell (2011) 144(5):646–74. doi: 10.1016/j.cell.2011.02.013 21376230

[11] Quail DF Joyce JA. Microenvironmental Regulation of Tumor Progression and Metastasis. Nat Med (2013) 19(11):1423–37. doi: 10.1038/nm.3394 PMC3954707 24202395

[12] Cleaver AL Beesley AH Firth MJ Sturges NC O’Leary RA Hunger SP. Gene-Based Outcome Prediction in Multiple Cohorts of Pediatric T-Cell Acute Lymphoblastic Leukemia: A Children’s Oncology Group Study. Mol Cancer (2010) 9:105. doi: 10.1186/1476-4598-9-105 20459861 PMC2879253

[13] Du W He J Zhou W Shu S Li J Liu W. High Il2ra Mrna Expression Is an Independent Adverse Prognostic Biomarker in Core Binding Factor and Intermediate-Risk Acute Myeloid Leukemia. J Transl Med (2019) 17(1):191. doi: 10.1186/s12967-019-1926-z 31171000 PMC6551869

[14] Fan T Pan S Yang S Hao B Zhang L Li D. Clinical Significance and Immunologic Landscape of a Five-Il(R)-Based Signature in Lung Adenocarcinoma. Front Immunol (2021) 12:693062. doi: 10.3389/fimmu.2021.693062 34497605 PMC8419226

[15] Barata JT Durum SK Seddon B. Flip the Coin: Il-7 and Il-7r in Health and Disease. Nat Immunol (2019) 20(12):1584–93. doi: 10.1038/s41590-019-0479-x 31745336

[16] Mazzucchelli RI Riva A Durum SK. The Human Il-7 Receptor Gene: Deletions, Polymorphisms and Mutations. Semin Immunol (2012) 24(3):225–30. doi: 10.1016/j.smim.2012.02.007 PMC6326362 22425228

[17] Kondo M Takeshita T Higuchi M Nakamura M Sudo T Nishikawa S. Functional Participation of the Il-2 Receptor Gamma Chain in Il-7 Receptor Complexes. Science (1994) 263(5152):1453–4. doi: 10.1126/science.8128231 8128231

[18] Al-Rawi MA Rmali K Watkins G Mansel RE Jiang WG. Aberrant Expression of Interleukin-7 (Il-7) and Its Signalling Complex in Human Breast Cancer. Eur J Cancer (2004) 40(4):494–502. doi: 10.1016/j.ejca.2003.10.016 14962714

[19] Ming J Zhang Q Qiu X Wang E. Interleukin 7/Interleukin 7 Receptor Induce C-Fos/C-Jun-Dependent Vascular Endothelial Growth Factor-D up-Regulation: A Mechanism of Lymphangiogenesis in Lung Cancer. Eur J Cancer (2009) 45(5):866–73. doi: 10.1016/j.ejca.2008.12.006 19136250

[20] Suzuki K Kadota K Sima CS Nitadori J Rusch VW Travis WD. Clinical Impact of Immune Microenvironment in Stage I Lung Adenocarcinoma: Tumor Interleukin-12 Receptor Beta2 (Il-12rbeta2), Il-7r, and Stromal Foxp3/Cd3 Ratio Are Independent Predictors of Recurrence. J Clin Oncol (2013) 31(4):490–8. doi: 10.1200/JCO.2012.45.2052 PMC3731922 23269987

[21] Alsadeq A Lenk L Vadakumchery A Cousins A Vokuhl C Khadour A. Il7r Is Associated with Cns Infiltration and Relapse in Pediatric B-Cell Precursor Acute Lymphoblastic Leukemia. Blood (2018) 132(15):1614–7. doi: 10.1182/blood-2018-04-844209 PMC6238156 30154115

[22] Kariminia A Ivison SM Leung VM Sung S Couto N Rozmus J. Y-Box-Binding Protein 1 Contributes to Il-7-Mediated Survival Signaling in B-Cell Precursor Acute Lymphoblastic Leukemia. Oncol Lett (2017) 13(1):497–505. doi: 10.3892/ol.2016.5437 28123588 PMC5244896

[23] Qin Y Zhu H Jiang B Li J Lu X Li L. Expression Patterns of Wt1 and Prame in Acute Myeloid Leukemia Patients and Their Usefulness for Monitoring Minimal Residual Disease. Leuk Res (2009) 33(3):384–90. doi: 10.1016/j.leukres.2008.08.026 18950857

[24] Zhu HH Jiang H Jiang Q Jia JS Qin YZ Huang XJ. Homoharringtonine, Aclarubicin and Cytarabine (Haa) Regimen as the First Course of Induction Therapy Is Highly Effective for Acute Myeloid Leukemia with T (8;21). Leuk Res (2016) 44:40–4. doi: 10.1016/j.leukres.2016.02.012 26994850

[25] Untergasser A Cutcutache I Koressaar T Ye J Faircloth BC Remm M. Primer3–New Capabilities and Interfaces. Nucleic Acids Res (2012) 40(15):e115. doi: 10.1093/nar/gks596 22730293 PMC3424584

[26] Gabert J Beillard E van der Velden VH Bi W Grimwade D Pallisgaard N. Standardization and Quality Control Studies of ‘Real-Time’ Quantitative Reverse Transcriptase Polymerase Chain Reaction of Fusion Gene Transcripts for Residual Disease Detection in Leukemia - a Europe against Cancer Program. Leukemia (2003) 17(12):2318–57. doi: 10.1038/sj.leu.2403135 14562125

[27] Qin YZ Zhu HH Jiang Q Xu LP Jiang H Wang Y. Heterogeneous Prognosis among Kit Mutation Types in Adult Acute Myeloid Leukemia Patients with T(8;21). Blood Cancer J (2018) 8(8):76. doi: 10.1038/s41408-018-0116-1 30087318 PMC6081455

[28] Qin YZ Li JL Zhu HH Li LD Chang Y Le H. Detection of Common Fusion Transcript Levels in Untreated Leukemia Patients by Real-Time Quantitative Rt-Pcr Technique. Zhonghua Xue Ye Xue Za Zhi (2007) 28(7):433–7. (Article in Chinese). 18072623

[29] Mazzucchelli R Durum SK. Interleukin-7 Receptor Expression: Intelligent Design. Nat Rev Immunol (2007) 7(2):144–54. doi: 10.1038/nri2023 17259970

[30] Sudo T Nishikawa S Ohno N Akiyama N Tamakoshi M Yoshida H. Expression and Function of the Interleukin 7 Receptor in Murine Lymphocytes. Proc Natl Acad Sci USA (1993) 90(19):9125–9. doi: 10.1073/pnas.90.19.9125 PMC47514 8415665

[31] Wei C Zeff R Goldschneider I. Murine Pro-B Cells Require Il-7 and Its Receptor Complex to up-Regulate Il-7r Alpha, Terminal Deoxynucleotidyltransferase, and C Mu Expression. J Immunol (2000) 164(4):1961–70. doi: 10.4049/jimmunol.164.4.1961 10657646

[32] Goldrath AW Sivakumar PV Glaccum M Kennedy MK Bevan MJ Benoist C. Cytokine Requirements for Acute and Basal Homeostatic Proliferation of Naive and Memory Cd8+ T Cells. J Exp Med (2002) 195(12):1515–22. doi: 10.1084/jem.20020033 PMC2193554 12070279

[33] Kieper WC Tan JT Bondi-Boyd B Gapin L Sprent J Ceredig R. Overexpression of Interleukin (Il)-7 Leads to Il-15-Independent Generation of Memory Phenotype Cd8+ T Cells. J Exp Med (2002) 195(12):1533–9. doi: 10.1084/jem.20020067 PMC2193553 12070281

[34] Tan JT Ernst B Kieper WC LeRoy E Sprent J Surh CD. Interleukin (Il)-15 and Il-7 Jointly Regulate Homeostatic Proliferation of Memory Phenotype Cd8+ Cells but Are Not Required for Memory Phenotype Cd4+ Cells. J Exp Med (2002) 195(12):1523–32. doi: 10.1084/jem.20020066 PMC2193564 12070280

[35] Schluns KS Kieper WC Jameson SC Lefrancois L. Interleukin-7 Mediates the Homeostasis of Naive and Memory Cd8 T Cells *in Vivo* . Nat Immunol (2000) 1(5):426–32. doi: 10.1038/80868 11062503

[36] Boyman O Purton JF Surh CD Sprent J. Cytokines and T-Cell Homeostasis. Curr Opin Immunol (2007) 19(3):320–6. doi: 10.1016/j.coi.2007.04.015 17433869

[37] Buentke E Mathiot A Tolaini M Di Santo J Zamoyska R Seddon B. Do Cd8 Effector Cells Need Il-7r Expression to Become Resting Memory Cells? Blood (2006) 108(6):1949–56. doi: 10.1182/blood-2006-04-016857 16705084

[38] Sinclair C Saini M Schim van der Loeff I Sakaguchi S Seddon B. The Long-Term Survival Potential of Mature T Lymphocytes Is Programmed During Development in the Thymus. Sci Signal (2011) 4(199):ra77. doi: 10.1126/scisignal.2002246 22087033

[39] Gattinoni L Klebanoff CA Restifo NP. Paths to Stemness: Building the Ultimate Antitumour T Cell. Nat Rev Cancer (2012) 12(10):671–84. doi: 10.1038/nrc3322 PMC6352980 22996603

[40] Mousset CM Hobo W Woestenenk R Preijers F Dolstra H van der Waart AB. Comprehensive Phenotyping of T Cells Using Flow Cytometry. Cytometry A (2019) 95(6):647–54. doi: 10.1002/cyto.a.23724 30714682

[41] Li Y Wu D Yang X Zhou S. Immunotherapeutic Potential of T Memory Stem Cells. Front Oncol (2021) 11:723888. doi: 10.3389/fonc.2021.723888 34604060 PMC8485052

[42] Xu L Yao D Tan J He Z Yu Z Chen J. Memory T Cells Skew toward Terminal Differentiation in the Cd8+ T Cell Population in Patients with Acute Myeloid Leukemia. J Hematol Oncol (2018) 11(1):93. doi: 10.1186/s13045-018-0636-y 29986734 PMC6038290

[43] Corrales L Matson V Flood B Spranger S Gajewski TF. Innate Immune Signaling and Regulation in Cancer Immunotherapy. Cell Res (2017) 27(1):96–108. doi: 10.1038/cr.2016.149 27981969 PMC5223230

[44] Spranger S Gajewski TF. Impact of Oncogenic Pathways on Evasion of Antitumour Immune Responses. Nat Rev Cancer (2018) 18(3):139–47. doi: 10.1038/nrc.2017.117 PMC6685071 29326431

[45] Barry M Bleackley RC. Cytotoxic T Lymphocytes: All Roads Lead to Death. Nat Rev Immunol (2002) 2(6):401–9. doi: 10.1038/nri819 12093006

[46] Ogawa M Matsuzaki Y Nishikawa S Hayashi S Kunisada T Sudo T. Expression and Function of C-Kit in Hemopoietic Progenitor Cells. J Exp Med (1991) 174(1):63–71. doi: 10.1084/jem.174.1.63 1711568 PMC2118893

[47] Turner AM Bennett LG Lin NL Wypych J Bartley TD Hunt RW. Identification and Characterization of a Soluble C-Kit Receptor Produced by Human Hematopoietic Cell Lines. Blood (1995) 85(8):2052–8. doi: 10.1182/blood.V85.8.2052.bloodjournal8582052 7536489

[48] Iiizumi S Ohtake J Murakami N Kouro T Kawahara M Isoda F. Identification of Novel Hla Class Ii-Restricted Neoantigens Derived from Driver Mutations. Cancers (Basel) (2019) 11(2):266. doi: 10.3390/cancers11020266 PMC6406322 30813491

