# Supplementary material for: Low IL7R Expression at Diagnosis Predicted Relapse in Adult Acute Myeloid Leukemia Patients With t(8;21)
Source: Front Immunol. 2022 Jul 7;13:909104. doi: 10.3389/fimmu.2022.909104 (PMC9302488; doi:10.3389/fimmu.2022.909104)
Supplement: Supplementary file 1 [file DataSheet_1.docx]

Supplementary Material

## 1. Supplementary Figures


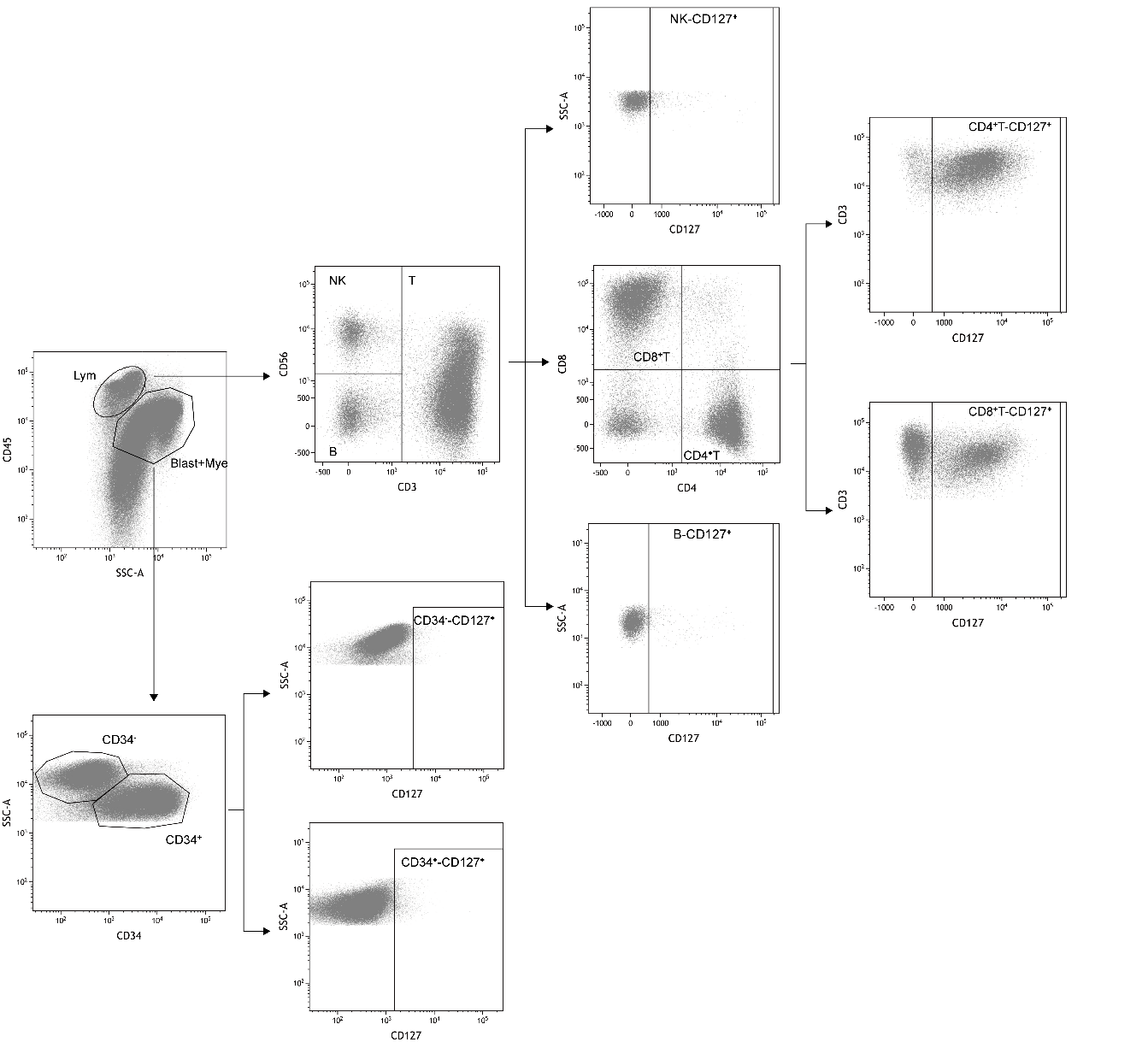


**Supplementary Figure 1.** Gating strategy for flow cytometry.
